# Effects of Different Sling Settings on Electromyographic Activities of Selected Trunk Muscles: A Preliminary Research

**DOI:** 10.1155/2020/2945952

**Published:** 2020-01-05

**Authors:** Xin Li, Howe Liu, Ke-yu Lin, Ping Miao, Bao-feng Zhang, Song-wei Lu, Le Li, Chu-huai Wang

**Affiliations:** ^1^Department of Rehabilitation Medicine, The First Affiliated Hospital, Sun Yat-sen University, Guangzhou 510080, China; ^2^Department of Physical Therapy, University of North Texas Health Science Center, Fort Worth, Texas 76101, USA; ^3^Department of Rehabilitation Medicine, The Second Affiliated Hospital of Guangzhou Medical University, Guangzhou 510260, China

## Abstract

**Introduction:**

The supine and prone sling exercise may facilitate activation of the local trunk muscles. Does the side-lying sling exercise activate trunk muscles more easily than the supine and prone training with sling settings? Clinical work has shown that the side-lying sling exercise could reduce pain in patients with unilateral low back pain (LBP), but the mechanism behind it is unclear. The fundamental purpose of this preliminary study was to examine the electromyography (EMG) characteristics of trunk muscles during different sling lumbar settings on sixteen healthy adults.

**Methods:**

Amplitude and mean power frequency (MPF) of EMG signals were recorded from the transversus abdominis (TA), rectus abdominis (RA), multifidus (MF), erector spinae (ES), gluteus maximus (Gmax), and gluteus medius (Gmed) muscles while the subjects performed the supine lumbar setting (SLS), prone lumbar setting (PLS), left side-lying lumbar setting (LSLS), and right side-lying lumbar setting (RSLS).

**Results:**

During SLS and PLS, TA and MF showed significantly higher activity than RA and ES on the same side, respectively. The EMG activities of ES, TA, MF, Gmax, and Gmed had significant differences between the different sides during LSLS and RSLS, and the dominant-side muscles showed higher activity than the other side. There was no significant difference in core trunk muscles between different sling lumbar settings—only that the SLS of the MF/ES ratio was significantly higher than LSLS and RSLS.

**Conclusions:**

Sling exercises can be an effective measure to enhance MF and TA EMG activity, and the side-lying position can increase dominant-side Gmax and Gmed activity. Side-lying sling training does not activate more core muscles than the supine and prone training. Supine and prone exercise should be preferred over SLT to stabilize the lumbar region because of its high local/global muscle ratio.

## 1. Introduction

Low back pain (LBP) has a high prevalence and can be an underlying cause of physical disability. As such, it should receive adequate attention. In a review of 165 studies from 54 countries concerning LBP, the mean point prevalence was estimated to be 18.3%, and 1-month prevalence was 30.8% [1]. LBP is related to decreased work ability and consequently increased direct (health care) and indirect (lost production and lost household productivity) costs. The etiology of LBP is still unknown, and it is not surprising that most current treatments for LBP are relatively ineffective [2]. The management of LBP is currently mainly based on the Panjabi theoretical model of the spinal stabilizing system [3]. Panjabi hypothesized that the neutral zone (NZ) is central to the phenomenon of instability and is critical to the range of motion extending beyond the NZ [4]. The NZ region is physiological, pain-free, and neurologically intact. However, when the NZ region increases with injury or weakness of the muscles, it may result in spinal instability or low-back problems [4]. These theoretical models may provide a universal system for body movement analysis and understanding of musculoskeletal disorders.

In recent decades, sling exercise has been used as an efficient training program for patients with musculoskeletal disorders [5–7]. This technique is a treatment method through which high levels of neuromuscular stimulation restore functional movement patterns [8]. It has four main elements. Firstly, the sling technique is a weight-bearing training that adjusts muscle coactivation to stabilize joints. Secondly, exercise intensity can be controlled through the elastic cords, which can unload body weight [9]. Thirdly, the sling technique provides uncontrolled stability by using the ropes and slings to increase proprioceptive inputs [10]. Finally, it is an efficient measure for adjusting postural balance, normalizing muscle response pattern, and reducing pain in patients with chronic LBP [5, 7, 8]. Studies have shown that in a supine position, a sling support surface can be used to significantly increase the thickness and activity of the transversus abdominis (TA) [11–13] in rehabilitation training programs for patients with LBP. Supine and prone bridge exercises can activate the multifidus (MF), rectus abdominis (RA), and erector spinae (ES) [14, 15]. In addition, the side-lying training (SLT) is one of the most commonly used exercises in rehabilitation to increase hip abduction [16–19] and trunk strength [20, 21]. Most previous studies focused on the optimization of movement between hip muscles (gluteus maximus (Gmax), gluteus medius (Gmed), and tensor fascia latae), which has been widely used in sports [16, 17] and clinical postoperative areas [19]. However, the effect of side-lying training (SLT) on the trunk muscles' activity has little research [20, 21]. Therefore, all of these may lead to another question: is the SLT (unilateral exercises) better for increasing greater muscular activation than supine and prone training (bilateral exercises) with sling settings?

Electromyography (EMG) has been widely used to study musculoskeletal system diseases, especially in core muscles [22]. Hodges and Richardson [23–25] studied the upper and lower limb movements using EMG and showed that TA (the deepest abdominal muscle) was the first trunk muscle to become active and is not specific to any direction. It was the only muscle found to be active prior to the anterior deltoid in the fast limb movement condition. EMG also showed that subjects who performed bridge exercises could activate the RA, MF, and ES muscles [10, 15]. Not only is core muscular dysfunction closely related to LBP, but Gmed weakness is also associated with LBP [11, 26–29]. The Gmed is a hip abductor and stabilizer of the pelvis; thus, increasing MF and Gmed activities could improve the lumbopelvic stability [26]. Hip function is an important contributor to both spine and trunk function; therefore, it likely plays a role in the development and response to LBP [21, 29]. Although these findings are suggestive of EMG activities in RA, TA, ES, MF, Gmax, or Gmed, it is still unclear if the effects of EMG activities of all of those trunk muscles would be different or similar with a variety of sling positions.

We aimed to clarify the EMG characteristics of RA, TA, ES, MF, Gmax, and Gmed during supine lumbar setting (SLS), prone lumbar setting (PLS), left side-lying lumbar setting (LSLS), and right side-lying lumbar setting (RSLS). We hypothesized that the selected trunk muscles would show different EMG activities with these SLS, PLS, LSLS, and RSLS positions.

## 2. Materials and Methods

### 2.1. Subjects

Sixteen healthy, young subjects in their 20s (8 males, 8 females) were recruited for this research, which was implemented in the hospital where the principal investigator worked. The mean age, weight, height, and body mass index (BMI) of the subjects were 20.6 ± 0.2 years, 58.1 ± 2.1 kg, 168.3 ± 2.2 cm, and 20.4 ± 0.5 kg/cm^2^, respectively. Inclusion criteria were as follows: BMI within ±20% of international standards; no musculoskeletal illnesses or previous or current neurological problems; no history of injury in the region of the abdomen, trunk, and lumbopelvic region and no scoliosis; no mental or psychological problems; must be right-handed. Participants were excluded if correct posture could not be maintained during exercises or female participants were menstruating or pregnant. The protocol was approved by the Human Subjects Ethics Subcommittee of the hospital (Approval number: [2017] C-034). All subjects gave written informed consent in accordance with the Declaration of Helsinki.

### 2.2. EMG Recording and Data Processing

The selected muscle activity was collected using a UMI-SE-I system (Shaoxing United Medical Instruments Co., Ltd., China) with 4-channel active electrodes (common mode rejection ratio of 110 db, bandwidth of 15–1000 Hz, a low noise <1 *μ*V, and resolution of 0.1 *μ*V). Sampling frequency from the EMG system was set to 3000 Hz and stored in the computer for analysis [30]. To reduce skin impedance, hair was removed from the measurement sites and the skin was cleaned with alcohol before electrode placement. Disposable Ag/AgCl surface electrodes were applied bilaterally to the muscles. The maximum spacing between the recording electrodes was 2.0 cm.  Table 1 shows the details of EMG electrode placement for the muscles under examination concerning the recommendation by SENIAM and the method applied in previous studies [14, 15, 28, 31].

There are numerous EMG parameters that can be used to describe muscle activity, including the most commonly used parameters like the frequency domain for the power spectral density and the time domain for the amplitude of the EMG signal. We analyzed the power spectrum with the parameter of mean power frequency (MPF) and the muscles' action potential with the maximum voluntary isometric contraction (MVIC). The measurement sites during the MVICs conforming to standardized muscle testing procedures are as follows [31]: RA/TA: let the spine flex by 30° and use a belt for supine position; ES/MF: extend the spine on a regular therapy table in the prone position; Gmax: it should be performed both in ∼20° hyperextension of hip and extended knee position with knee extended in the prone position; Gmed: hip abduction was performed as far as possible in a fixated side-lying position. For each MVIC measured, those lasting 5 seconds were performed 3 times and the highest was then selected. There was 1 minute of rest between each MVIC. In order to help motivate subjects to achieve maximal muscle activity, verbal encouragement was used. The average EMG of the 3 replicates was used in the analysis after normalization to %MVIC [5–7, 15], and mean power frequency (MPF) was also recorded. To minimize muscle fatigue, a 1-minute break was taken after each muscle. All of the 6 assessed muscles were tested bilaterally, and the muscles of the abdomen, lower back, and buttocks were tested separately. In order to analyze relative values of muscular activities for postural stabilization (MF and TA) to those of muscles for postural movement (ES and RA), the MF/ES and TA/RA ratios were calculated for further analysis.

### 2.3. Procedures

**Figure 1:**
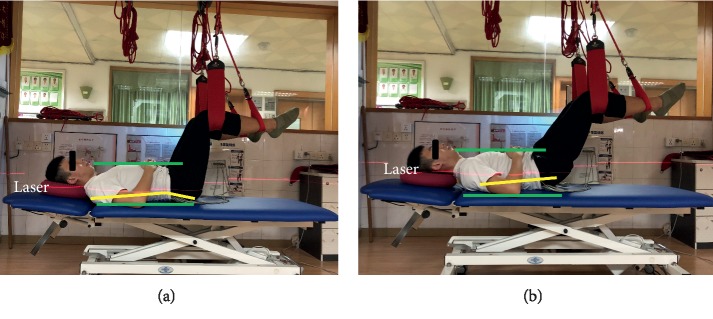
SLS performed.

**Figure 2:**
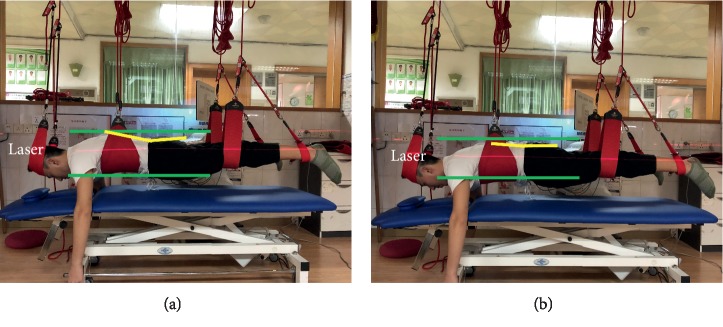
PLS performed.

**Figure 3:**
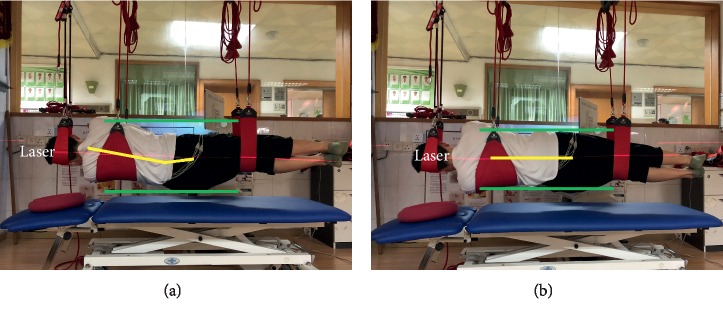
LSLS/RSLS performed.

Four types of sling lumbar settings were performed as follows (Table 2). They were performed in random order, which was determined by selecting a single card with four cards marked with either 1 (SLS), 2 (PLS), 3 (LSLS), or 4 (RSLS). The investigator helped participants place their pelvis in a neutral position (Figures 1(b), 2(b), and 3(b)). A laser (red line) was used to provide a beam to ensure the levelled/controlled position. The upper and lower green lines help to identify the ventral and dorsal side of the pelvis, respectively. The yellow line helps to estimate the angle between the sacral vertebrae and the trunk. The neutral position was achieved when the red line was parallel or coincident with the yellow line [12]. They were instructed to hold the sling setting for 10 s without focusing on contracting specific muscles. Each exercise was performed 3 times, and a 2-minute break was taken between each exercise. The middle 5 seconds of data, after discarding the first three seconds and last two seconds, were used in the analysis. The average EMG of the 3 replicates was used in the analysis [15]. In order to help motivate subjects to maintain the sling settings, verbal encouragement was used. No subjects developed fatigue or were unable to maintain the standard 10-second sling lumbar settings.

### 2.4. Data Analysis

Statistical Package for the Social Sciences version 25.0 software (SPSS Inc., Chicago, IL, USA) was used for statistical analysis of all results. All EMG data were expressed as means with standard error. The Shapiro–Wilk test was used to test the normality distribution of the data. As normality was accepted, the paired *t*-test was used to determine the two sides and the same side of muscle activity. Wilcoxon's signed-rank test was used for nonnormal distribution parameters. ANOVA compared the dominant-side local trunk muscles (MF and TA) and relative EMG activity ratios (MF/ES and TA/RA) among the four sling lumbar settings. If a significant difference was found, then a post hoc test was performed using the Bonferroni correction, and the adjusted *p* value was set at *p* < 0.0083. Statistical significance level was set at *p* < 0.05.

## 3. Results

### 3.1. EMG Activity (%MVIC)

**Figure 4:**
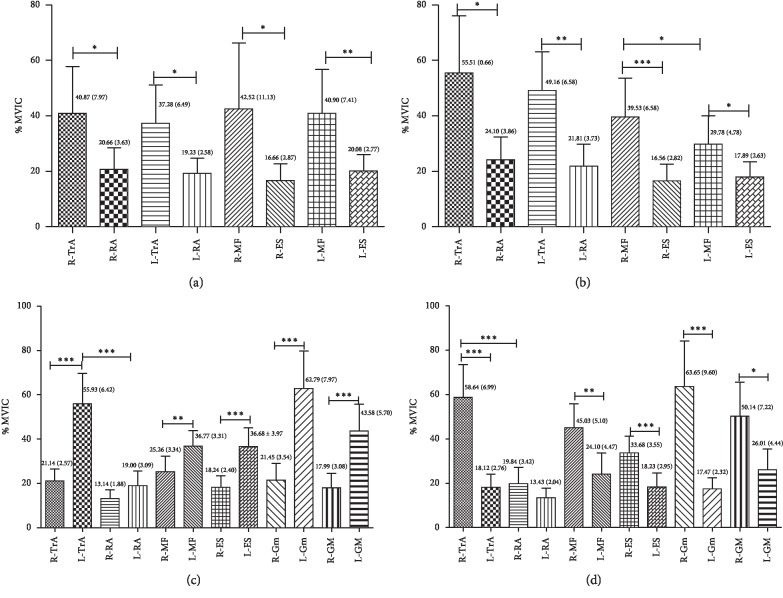
Comparisons of %MVIC between the same side and different side of local trunk muscle and global trunk muscle (TA and RA; MF and ES), Gmax, and Gmed in the same sling lumbar settings. R: right; L: left; TA: transversus abdominis; RA: rectus abdominis; MF: multifidus; ES: erector spinae; Gmax: gluteus maximus; Gmed: gluteus medius; SLS: supine lumbar setting; PLS: prone lumbar setting; LSLS: left side-lying lumbar setting; RSLS: right side-lying lumbar setting. ^*∗*^*p* < 0.05; ^*∗*^^*∗*^*p* < 0.01; ^*∗∗∗*^*p* < 0.001. (a) SLS. (b) PLS. (c) LSLS. (d) RSLS.

Figure 4 describes %MVIC data collected for TA, RA, MF, ES, Gmax, and Gmed while performing four lumbar settings, respectively, in the same muscle of different sides and different muscles of the same side with the same sling lumbar settings. At the SLS and PLS positions (Figures 4(a) and 4(b)), the same muscle had no significant differences between the left and right sides (*p* > 0.05) except the MF on the right side of the PLS position (*p*=0.042; Figure 4(b)). On the same side, both TA and MF showed greater EMG activity during SLS (R: TA: *p*=0.044 and MF: *p*=0.026; L: TA: *p*=0.022 and MF: *p*=0.006; Figure 4(a)) and PLS (R: TA: *p*=0.018 and MF: *p* < 0.001; L: TA: *p*=0.007 and MF: *p*=0.018; Figure 4(b)) compared to RA and ES, respectively. In the pairwise analysis of LSLS and RSLS (Figures 4(c) and 4(d)), the dominant-side (in the RSLS position, right side is the dominant side) muscles showed greater EMG activity than the nondominant side (*p* < 0.05). Comparison on the same side showed that the TA had greater EMG activity than the RA (LSLS: *p* < 0.001, Figure 4(c); RSLS: *p* < 0.001, Figure 4(d)), both only on the dominant side. The Gmed showed no significant difference versus Gmax in LSLS and RSLS.

### 3.2. MPF

**Figure 5:**
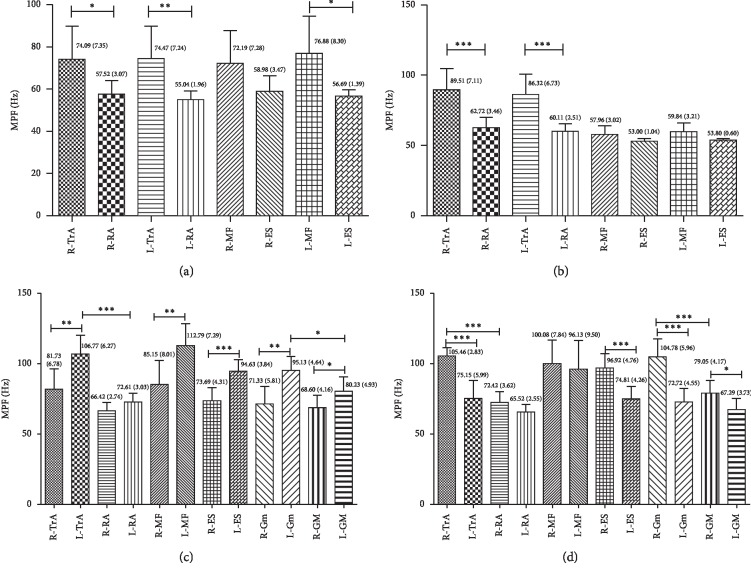
Comparisons of MPF between the same side and different side of local trunk muscle and global trunk muscle (TA and RA; MF and ES), Gmax, and Gmed with the same sling lumbar settings. R: right; L: left; TA: transversus abdominis; RA: rectus abdominis; MF: multifidus; ES: erector spinae; Gmax: gluteus maximus; Gmed: gluteus medius; SLS: supine lumbar setting; PLS: prone lumbar setting; LSLS: left side-lying lumbar setting; RSLS: right side-lying lumbar setting. ^*∗*^*p* < 0.05; ^*∗∗*^*p* < 0.01; ^*∗∗∗*^*p* < 0.001. (a) SLS. (b) PLS. (c) LSLS. (d) RSLS.

Figure 5 describes MPF data collected for TA, RA, MF, ES, Gmax, and Gmed while performing four lumbar settings, respectively. At the SLS and PLS positions of the same side, TA showed greater EMG activity during SLS (R: TA: *p*=0.019; L: TA: *p*=0.009; Figure 5(a)) and PLS (R: TA: *p*=0.001; L: TA: *p* < 0.001; Figure 5(b)) compared to RA, respectively. MF showed no significant differences compared to ES (*p* > 0.05) except the left side of the SLS position (*p*=0.019; Figure 5(a)). In the pairwise analysis of both LSLS (Figure 5(c)) and RSLS (Figure 5(d)), the dominant-side muscles showed greater MPF than the other side (*p* < 0.05). For the RA, no difference in MPF was found between LSLS (*p*=0.107; Figure 5(c)) and RSLS (*p*=0.106; Figure 5(d)), and for TA, no significant difference was found in RSLS (*p*=0.622; Figure 5(d)). At the same side, the dominant-side TA and Gmed both showed greater MPF compared to RA (LSLS: *p* < 0.001, Figure 5(c); RSLS: *p* < 0.001, Figure 5(d)) and Gmax (LSLS: *p* < 0.023; Figure 5(c); RSLS: *p*=0.001; Figure 5(d)).

### 3.3. TA and MF

**Figure 6:**
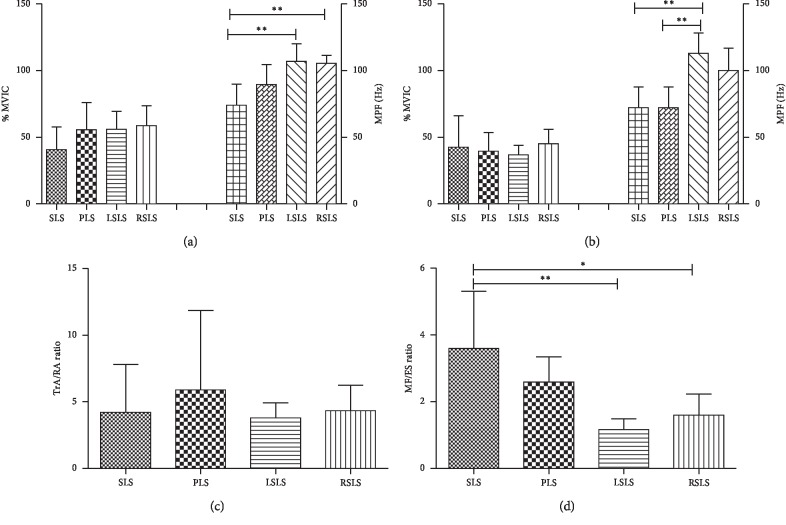
Multiple comparisons of (a) TA, (b) MF, (c) TA/RA, and (d) MF/ES in different Neurac lumbar settings. TA: transversus abdominis; RA: rectus abdominis; MF: multifidus; ES: erector spinae; SLS: supine lumbar setting; PLS: prone lumbar setting; LSLS: left side-lying lumbar setting; RSLS: right side-lying lumbar setting. ^*∗*^*p* < 0.05; ^*∗*^^*∗*^*p* < 0.01; ^*∗∗∗*^*p* < 0.001.

Figure 6 shows no difference in the local trunk muscles (TA: *F* = 1.045, *p*=0.379, Figure 6(a); MF: *F* = 0.252, *p*=0.860, Figure 6(b)) between different sling lumbar settings. The TA/RA ratios showed no statistically significant (*F* = 0.292, *p*=0.831; Figure 6(c)) difference. However, the MF/ES ratios showed a significant difference (*F* = 5.272, *p*=0.003; Figure 6(d)) between the four types of lumbar settings. Figure 6(d) shows that the SLS of the MF/ES ratios was significantly higher than LSLS (*p*=0.003) and RSLS (*p*=0.024).

## 4. Discussion

### 4.1. Summary of Our Results

Our results suggested that the activations of MF and TA—which contribute to lumbar region stabilization during everyday activities—were significantly higher than those of RA and ES, respectively (Figure 4). There was no significant difference in EMG activity between Gmax and Gmed, but the dominant-side TA and Gmed both showed greater MPF compared to RA and Gmax (Figure 5). We found no evidence to support our hypothesis that the core muscles were activated differently in different positions—only that the SLS of the MF/ES ratios was significantly higher than LSLS and RSLS and there are significant differences in MPF (Figure 6). The analysis focused on the relationship between the values of MPF and %MVC with consideration of confounding factors, such as muscle length, group of subjects, the electrodes, and type of contraction [32]. One possible explanation for discrepancies seen may be related to the limited number of healthy, young participants, who had a greater sensitivity to alter spontaneous neuronal activity resulting in EMG activity changes and muscle performance. Moreover, subjects with a BMI ±20% were used to ensure optimal EMG recordings and avoid adipose tissue signal affecting amplitude and frequency. A second explanation may be related to the EMG data measured for 5 seconds. This is just an immediate effect, not a long-term effect. In future study, a longer recording time of 30 s might be performed to observe the effect of fatigue between posture and EMG activity.

### 4.2. Comparison of Our Findings with Previous Studies for the Position of SLS

Previous literature has many different studies on supine bridging exercises. Based on the numerical values of EMG, the current studies stated that the bilateral unstable supine plank was the most challenging exercise to enhance MF EMG activity among stable, unstable, and unilateral bridging exercises [33, 34]. However, the participants in these studies performed the position with the knees fully extended, but our study used the Lewit position, which provides different body positions and unstable conditions. The Lewit exercise was performed with the subject lying supine in a crook-lying position (as shown in Figure 1) with the hips flexed to form two 90° angles between the thigh and the trunk as well as between the thighs and legs. The Lewit exercise caused higher muscle activity in the deeper abdominal wall muscles, particularly TA [35]. Our findings also supported previous studies that SLS helps reinforce the activity of the TA compared to the global trunk muscle of RA (Figure 4(a)). Previous results [33] suggested that none of the bilateral exercises (stable or unstable) produced side-to-side differences for MF, which appeared to contradict our findings. This could be because of using a less stable position for unsupported limbs in these previous studies. Here, the Lewit exercise was a relatively more stable posture. In addition, this research did not compare the relative activity level of the local trunk-stabilizing muscles to that of the larger global muscles [33], which our research does. Spinal pain can result in atrophy of local trunk-stabilizing muscles. Understanding which muscle activity contributes to trunk stabilization during daily activities can help clinicians address these deficits [3, 36].

### 4.3. Comparison of Our Findings with Previous Studies for the Position of LSLS/RSLS

Although side-lying bridging exercises are commonly prescribed for patients with LBP, few studies have provided valuable information to support treatment interventions for targeting specific trunk muscles—especially side-lying sling exercises [7, 37]. A recent study reported that the pelvic compression belt and manual pelvic fixation increase the Gmed and MF, while decreasing quadratus lumborum prevents unwanted substitution movement during side-lying hip abduction [20, 21]. Our results found that side-lying lumbar setting could improve not only the activity of MF and Gmed but also the activity of TA and Gmax; these results were partially consistent with those of Park and colleagues [20]. Our findings show that the opposite side muscles were activated (Figures 4(c) and 4(d)). Our study improved the dominant-side (bottom) muscles rather than the other (top) side muscles because the sling lumbar setting programs were mostly in the form of a closed kinetic chain exercise. Weakness of hip abductors impacts the optimal force closure mechanism via the sacroiliac joints; this might cause dysfunction of deep core muscles such as MF [38]. Due to its mechanical and functional benefits, the side-lying position is frequently used in the rehabilitation of individuals with lower back and hip pathologies. Clinically, many patients with LBP have asymmetrical symptoms. This study provides available information to supply treatment interventions for targeting specific trunk and abdomen muscles or specific unilateral atrophy within the LBP population.

### 4.4. View of Lumbar Stabilization Training and Clinical Value

The normal function of the stabilizing system provides sufficient stability for the spine to match the instantaneously varying stability demands due to changes in spinal posture, as well as the static and dynamic loads. The three subsystems consisting of the neural subsystem, spinal column, and spinal muscles work together to achieve this goal [3]. As our study shows, TA and MF muscles under different lumbar settings are more active than RA and ES muscles, but RA and ES muscles also contribute to lumbar stabilization (Figure 4). Stability and coordination training between muscles (the active subsystem) are always our intervention for patients. As mentioned earlier, sling exercises are now performed for a wide range of patients with musculoskeletal problems including those with LBP, athletes, etc. Although our research focused on the role of muscles for postural stabilization in lumbar stabilization, the importance of coactivation of muscular systems for maintaining lumbar stability has been recognized in clinical knowledge. Moreover, the modern medical model puts more emphasis on holistic medicine [2]. The coordinated operation of each system can make the body play a better role and improve performance in sports [3, 4]. Our findings that single muscles were not activated differently in different positions may support these points (Figure 4). In addition, our result shows that SLS and PLS have high MF/ES ratios (Figures 6(c) and 6(d)), which implies an improvement in muscle activity of local muscles. Relatively difficult tasks, such as LSLS and RSLS, have high muscle activity in the overall muscles but should be avoided when training local muscle strengthening due to low MF/ES ratios. So SLS and PLS are the preferred positions to stabilize the lumbar region.

### 4.5. Limitations and Strengths

Our study has several limitations. Firstly, this is a preliminary research focusing on investigating EMG activities of trunk muscles under different sling settings, and we recruited a relatively small group of healthy, young individuals. Therefore, our findings may be hard to generalize for other populations with lumbopelvic disorders such as sacroiliac joints dysfunction or low back pain. In addition, cross talk from adjacent muscles is present in every EMG collection, although we have adopted measures of skin preparation and electrode placement to prevent this. Lastly, the protocol of the current study could not test muscle fatigue because the collection time was only ten seconds. In our future study, we will extend the collection time to study muscle fatigue in various lumbar sling settings. Nevertheless, this study offers main suggestions for training programs and increasing the knowledge about possible progressions or variations to improve muscle function and lumbar stability. For instance, an effective trunk training progression could integrate lumbar settings as the first step. Alternatives include targeting specific trunk muscles within a definite diagnosis or treating the dominant side where there may be unilateral muscle imbalances. Therefore, our future research will apply lumbar sling settings to patients with LBP to see if it can improve their pain and function.

## 5. Conclusions

The present study shows that sling exercise may facilitate activation of the local trunk muscles, such as MF and TA and even the pelvic stabilizers Gmax and Gmed, and that SLT does not activate more core muscles than the supine and prone training during sling settings. In addition, supine and prone exercise should be preferred over SLT to stabilize the lumbar region because of its high local/global muscle ratio. These exercises are feasible, relatively efficacious, and easily applied in the clinic. Thus, our findings provide useful information to clinical evaluation and the design of rehabilitation intervention for optimum functional recovery of LBP.

## Figures and Tables

**Table 1:** Placement of the EMG electrodes.

Muscle	Electrode placement location
Transversus abdominis (TA)	Along either side of the course of the underlying muscle fibers and centered 2 cm cephalic to the pubic bone, just lateral to the midline, and parallel to the superior pubic ramus [15]

Rectus abdominis (RA)	2 cm lateral from the midline of the umbilicus [15]

Multifidus (MF)	A line from caudal tip posterior spinal iliac superior to the interspace between L1 and L2 interspace at the level of L5 spinous process (i.e., about 2-3 cm from the midline) [15]

Erector spinae (ES)	2 cm lateral to the L3 level [31]

Gluteus medius (Gmed)	1/2 on the line from the highest point of the iliac crest to the greater trochanter [28]

Gluteus maximus (Gmax)	1/2 on the line between the sacral vertebrae and the greater trochanter; this position corresponds to the greatest prominence in the middle of the buttocks above the visible bulge of the greater trochanter [14]

**Table 2:** Four types of sling lumbar settings.

Position	Exercise method
SLS (Figure 1(a))	Subject supine with arms on the abdomen. Elevate legs to 90 degrees of hip and knee flexion. Lower treatment table to lift the pelvis from the surface.

PLS (Figure 2(a))	Subject prone with wide sling under the chest. Narrow slings distally on the thighs. The head supported in a split sling, attached with black elastic cords to rope in the pulley.

LSLS/RSLS (Figure 3(a))	Subject side-lying with wide sling under the chest and hands on the chest. Narrow sling distally on the thighs. The head supported in a split sling attached with black elastic cords to rope in the pulley. The left side is the dominant side in LSLS and vice versa.

SLS: supine lumbar setting; PLS: prone lumbar setting; LSLS: left side-lying lumbar setting; RSLS: right side-lying lumbar setting.

## Data Availability

The data used to support the findings of this study are available from the corresponding author upon request.

## References

[1] Hoy D., Bain C., Williams G. (2012). A systematic review of the global prevalence of low back pain. *Arthritis & Rheumatology*.

[2] Chris M., Martin U., Rachelle B. (2017). Non-specific low back pain. *The Lancet*.

[3] Panjabi M. M. (1992). The stabilizing system of the spine: part I. Function, dysfunction, adaptation, and enhancement. *Journal of Spinal Disorders*.

[4] Panjabi M. M. (1992). The stabilizing system of the spine. Part II. Neutral zone and instability hypothesis. *Journal of Spinal Disorders*.

[5] Lee S. W., Kim S. Y. (2015). Effects of hip exercises for chronic low-back pain patients with lumbar instability. *Journal of Physical Therapy Science*.

[6] Linek P., Saulicz E., Myśliwiec A., Wójtowicz M., Wolny T. (2015). The effect of specific sling exercises on the functional movement screen score in adolescent volleyball players: a preliminary study. *Journal of Human Kinetics*.

[7] Park H., Jeong T., Lee J. (2017). Effects of sling exercise on flexibility, balance ability, body form, and pain in patients with chronic low back pain. *Rehabilitation Nursing*.

[8] Kim J. H., Kim Y. E., Bae S. H., Kim K. Y. (2013). The effect of the neurac sling exercise on postural balance adjustment and muscular response patterns in chronic low back pain patients. *Journal of Physical Therapy Science*.

[9] Yun K., Lee S., Park J. (2015). Effects of closed chain exercises for the lumbar region performed with local vibration applied to an unstable support surface on the thickness and length of the transverse abdominis. *Journal of Physical Therapy Science*.

[10] Choi Y., Kang H. (2013). The effects of sling exercise using vibration on trunk muscle activities of healthy adults. *Journal of Physical Therapy Science*.

[11] Eom M. Y., Chung S. H., Ko T. S. (2013). Effects of bridging exercise on different support surfaces on the transverse abdominis. *Journal of Physical Therapy Science*.

[12] Jörn L., Kim J. B., Christian P., Tobias L. S., Heiko W. (2015). Using ultrasound to assess the thickness of the transversus abdominis in a sling exercise. *BMC Musculoskeletal Disorders*.

[13] Lee D., Park J., Lee S. (2015). Effects of bridge exercise on trunk core muscle activity with respect to sling height and hip joint abduction and adduction. *Journal of Physical Therapy Science*.

[14] Choi S.-A., Cynn H.-S., Yi C.-H. (2015). Isometric hip abduction using a thera-band alters gluteus maximus muscle activity and the anterior pelvic tilt angle during bridging exercise. *Journal of Electromyography and Kinesiology*.

[15] Park H.-J., Oh D.-W., Kim S.-Y. (2014). Effects of integrating hip movements into bridge exercises on electromyographic activities of selected trunk muscles in healthy individuals. *Manual Therapy*.

[16] Mcbeth J. M., Earl-Boehm J. E., Cobb S. C., Huddleston W. E. (2012). Hip muscle activity during 3 side-lying hip-strengthening exercises in distance runners. *Journal of Athletic Training*.

[17] Bishop B. N., Greenstein J., Etnoyer-Slaski J. L., Sterling H., Topp R. (2018). Electromyographic analysis of gluteus maximus, gluteus medius, and tensor fascia latae during therapeutic exercises with and without elastic resistance. *International Journal of Sports Physical Therapy*.

[18] Thorborg K., Bandholm T., Petersen J. (2010). Hip abduction strength training in the clinical setting: with or without external loading?. *Scandinavian Journal of Medicine & Science in Sports*.

[19] Pauley T., Devlin M., Madan-Sharma P. (2014). A single-blind, cross-over trial of hip abductor strength training to improve timed up & go performance in patients with unilateral, transfemoral amputation. *Journal of Rehabilitation Medicine*.

[20] Kim E. H., Lim T. H., Park S. H. (2015). Effect of hip abduction exercise with manual pelvic fixation on recruitment of deep trunk muscles. *American Journal of Physical Medicine & Rehabilitation*.

[21] Park K.-M., Kim S.-Y., Oh D.-W. (2010). Effects of the pelvic compression belt on gluteus medius, quadratus lumborum, and lumbar multifidus activities during side-lying hip abduction. *Journal of Electromyography and Kinesiology*.

[22] du Rose A. (2018). Have studies that measure lumbar kinematics and muscle activity concurrently during sagittal bending improved understanding of spinal stability and sub-system interactions? A systematic review. *Healthcare*.

[23] Hodges P. W., Richardson C. A. (1997). Relationship between limb movement speed and associated contraction of the trunk muscles. *Ergonomics*.

[24] Hodges P. W., Richardson C. A. (1997). Contraction of the abdominal muscles associated with movement of the lower limb. *Physical Therapy*.

[25] Hodges P. W., Richardson C. A. (1998). Delayed postural contraction of transverusus abdominis in low back pain associated with movement of the lower limb. *Archives of Physical Medicine and Rehabilitation*.

[26] Cooper N. A., Scavo K. M., Strickland K. J. (2016). Prevalence of gluteus medius weakness in people with chronic low back pain compared to healthy controls. *European Spine Journal*.

[27] Nelson-Wong E., Gregory D. E., Winter D. A., Callaghan J. P. (2008). Gluteus medius muscle activation patterns as a predictor of low back pain during standing. *Clinical Biomechanics*.

[28] Bewyer K. J., Bewyer D. C., Messenger D. (2009). Pilot data: association between gluteus medius weakness and low back pain during pregnancy. *The Iowa Orthopaedic Journal*.

[29] Bussey M. D., Kennedy J. E., Kennedy G. (2016). Gluteus medius coactivation response in field hockey players with and without low back pain. *Physical Therapy in Sport*.

[30] Zhang S. S., Xu Y., Han X. L., Wu W., Tang Y., Wang C. (2018). Functional and morphological changes in the deep lumbar multifidus using electromyography and ultrasound. *Scientific Reports*.

[31] Konrad P. (2005). *ABC of EMG—A Practical Introduction to Kinesiological Electromyography*.

[32] Roman-Liu D. (2016). The influence of confounding factors on the relationship between muscle contraction level and MF and MPF values of EMG signal: a review. *International Journal of Occupational Safety and Ergonomics*.

[33] Feldwieser F. M., Sheeran L., Meana-Esteban A., Sparkes V. (2012). Electromyographic analysis of trunk-muscle activity during stable, unstable and unilateral bridging exercises in healthy individuals. *European Spine Journal*.

[34] Calatayud J., Casaña J., Martín F. (2017). Trunk muscle activity during different variations of the supine plank exercise. *Musculoskeletal Science and Practice*.

[35] Badiuk B. W. N., Andersen J. T., McGill S. M. (2014). Exercises to activate the deeper abdominal wall muscles. *Journal of Strength and Conditioning Research*.

[36] Kliziene I., Sipaviciene S., Klizas S., Imbrasiene D. (2015). Effects of core stability exercises on multifidus muscles in healthy women and women with chronic low-back pain. *Journal of Back and Musculoskeletal Rehabilitation*.

[37] Unsgaard-Tøndel M., Fladmark A. M., Salvesen Ø., Vasseljen O. (2010). Motor control exercises, sling exercises, and general exercises for patients with chronic low back pain: a randomized controlled trial with 1-year follow-up. *Physical Therapy*.

[38] Pool-Goudzwaard A. L., Vleeming A., Stoeckart R., Snijders C. J., Mens J. M. A. (1998). Insufficient lumbopelvic stability: a clinical, anatomical and biomechanical approach to “a-specific” low back pain. *Manual Therapy*.

